# Integrated analysis of incidence, progression, regression and disappearance probabilities

**DOI:** 10.1186/1471-2288-8-40

**Published:** 2008-06-25

**Authors:** Guan-Hua Huang

**Affiliations:** 1 Institute of Statistics, National Chiao Tung University, Hsinchu, Taiwan

## Abstract

**Background:**

Age-related maculopathy (ARM) is a leading cause of vision loss in people aged 65 or older. ARM is distinctive in that it is a disease which can transition through incidence, progression, regression and disappearance. The purpose of this study is to develop methodologies for studying the relationship of risk factors with different transition probabilities.

**Methods:**

Our framework for studying this relationship includes two different analytical approaches. In the first approach, one can define, model and estimate the relationship between each transition probability and risk factors separately. This approach is similar to constraining a population to a certain disease status at the baseline, and then analyzing the probability of the constrained population to develop a different status. While this approach is intuitive, one risks losing available information while at the same time running into the problem of insufficient sample size. The second approach specifies a transition model for analyzing such a disease. This model provides the conditional probability of a current disease status based upon a previous status, and can therefore jointly analyze all transition probabilities. Throughout the paper, an analysis to determine the birth cohort effect on ARM is used as an illustration.

**Results and conclusion:**

This study has found parallel separate and joint analyses to be more enlightening than any analysis in isolation. By implementing both approaches, one can obtain more reliable and more efficient results.

## Background

The present paper was motivated by an earlier population-based longitudinal study of age-related ocular disorders. Here, we focus on age-related maculopathy (ARM), a leading cause of vision loss in the elderly. ARM is characterized by the distinctive "transition" property: once the incident occurs, the disease can progress, regress, and disappear. This transition characteristic is also exhibited by several other diseases [1-3]. Traditional statistical methods provide information on the risk of "having a disease" (prevalence). The analysis of the transition course of ARM poses a challenge. The purpose of our study is to develop a methodology for studying the relationship between risk factors and an individual's disease transition, including incidence, progression, regression and disappearance.

If we classify a change in the severity of the disease by defining a three-level scale: disease-free, early and late stage, then different transition courses can be defined as the current disease level conditioning upon the level at the immediately preceding examination. Incidence of the disease implies the appearance of the disease at the current examination when it was absent at the preceding examination. Progression implies that an individual is initially diagnosed with an early stage of the disease with worsening at the current examination, while regression implies the presence of the disease at the preceding examination with an improvement at the current examination. Disappearance implies the presence of the disease at the preceding examination and its absence at the current examination. Because of the nature of the definition, an obvious way to analyze the data is to constrain the study population to individuals with a specific disease level at the initial examination. We can then analyze the probability of the constrained population developing a different level at follow-up. The choice of the disease level will then depend on the type of transition we are interested in, and each type of transition can be analyzed separately. For example, when studying progression, we will include only those individuals that are classified as being in the early stage in the initial exam in our analysis. We then study the probability of developing a late stage of the disease at follow-up.

While this approach is intuitive, we risk losing some of our available information. For example, let's look at a study in which each participant is measured at the baseline and at 5-year and 10-year follow-up examinations. A disease must be present at the 5-year follow-up for progression to be possible at the 10-year follow-up, therefore, the incidence of a disease at the 5-year examination and its progression at the 10-year examination are correlated. By separating incidence and progression, we waste the valuable correlation between two transitions. We may also encounter the difficulty of an insufficient sample size. For the "rare" disease where only a small number of cases are observed, the study population for progression, regression and disappearance probabilities will be small. A model with many covariates of interest may not converge due to an insufficient sample size.

An alternative approach is based on a transition model. The model assumes that there is a correlation among repeated measurements because the past values explicitly influence the present observation. It formulates the conditional distribution of each measurement as a function of past observations and relevant risk factors. The transition model provides the conditional probability of a current disease level based upon its previous level. This is one way we can define the incidence, progression, regression and disappearance probabilities. By joint analysis, this approach takes the correlations among various transition probabilities into account and allows some confounding variables to have an equal effect on various transition probabilities, which in turn can ease the problem of insufficient sample size described above. However, these benefits come at the price of stronger modelling assumptions.

The remainder of this paper is organized as follows. In the methods section, we first briefly describe the research project that motivated this study and define the distinct transition probabilities of ARM. Next, we summarize the approach for analyzing the transition probabilities separately, and then we introduce a transition model to analyze them jointly. In addition we discuss parameter interpretation and estimation. Finally, we show how separate and joint analyses can be used together to obtain more reliable and efficient results. The results section applies our methodology to analyze the birth cohort effect on different transition probabilities of ARM, and we discuss the possible generalization of the proposed model.

## Methods

### The Beaver Dam Eye Study

The Beaver Dam Eye Study, a longitudinal cohort study of residents of Beaver Dam, Wisconsin between the ages of 43 and 84 years in 1987–1988, has been described in detail elsewhere [4-6]. This study aims to determine the long-term course of common vision-threatening conditions in adult Americans. The 4,926 individuals that participated in the baseline examination in 1988–1990, decreased to 3,684 at the 5-year follow-up in 1993–1995 due to death, relocation or refusal, then decreased to 2,764 at the 10-year follow-up in 1998–2000, and then further decreased to 2,119 at the 15-year follow-up in 2003–2005. Drop-outs were older and less educated than those who participated in the follow-up examinations. There were no other statistically significant differences while controlling for age [5,6].

### ARM severity scale and transition probabilities

**Figure 1:**
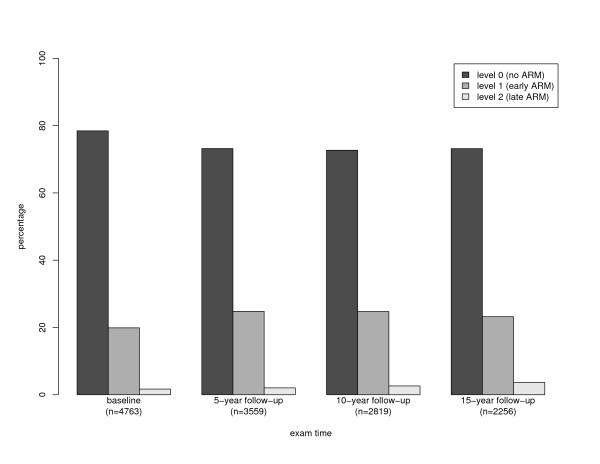
**Proportions of different severity levels**. Proportions of different levels of ARM severity scale in the worst eye at baseline, 5-year follow-up, 10-year and 15-year follow-up: Beaver Dam Eye Study (1988–2005). n = the number of participants whose ARM severity measurements are available at that time point.

Procedures for obtaining and evaluating photographs of participants' eyes have been described elsewhere [4]. At each examination, 30 degree color stereoscopic fundus photographs were taken of both eyes of each participant. Two gradings (preliminary and detailed) were performed for each eye at each examination. Next, a series of edits and reviews was performed, and standardized edit rules were used to adjudicate any disagreements. As a result of this edit, only a few changes were made [6]. The grading used the fundus photographs to determine the severity of the ARM lesions, which were graded on a 6-level scale [7]. For this study, the scale was collapsed to three levels in order of increasing severity: level 0 = disease free, level 1 = early ARM, and level 2 = late ARM. The results presented here use each individual's ARM level in the eye with the worst condition. Proportions for different levels in the worse eye at baseline, 5-year follow-up, 10-year follow-up and 15-year follow-up are shown in Figure 1.

We define a transition course of ARM as the current ARM level conditioning after the preceding level, as described in the background section. Probabilities of different courses can be represented in the form of conditional probability and are defined in Table 1. It should be noted that we have treated the transition of level 2 to level 0 as a regression rather than a disappearance. This was done to make the results from separate and joint analyses comparable. Due to some modeling limitations, the regression and disappearance probabilities cannot be simultaneously estimated by the transition model when based on the more desirable definition altering the 2-to-0 transition to affiliate with disappearance. In the discussion section, we have provided details on how this affects our result and what possible modification can be made.

**Table 1:** Definitions of distinct ARM transition probabilities

probabilities/time	baseline	5-year	10-year	15-year
prevalence	Pr(ARM(0) = 1,2)	Pr(ARM(5) = 1,2)	Pr(ARM(10) = 1,2)	Pr(ARM(15) = 1,2)
incidence	N/A	Pr(ARM(5) = 1,2|ARM(0) = 0)	Pr(ARM(10) = 1,2|ARM(5) = 0)	Pr(ARM(15) = 1,2|ARM(10) = 0)
progression	N/A	Pr(ARM(5) = 2|ARM(0) = 1)	Pr(ARM(10) = 2|ARM(5) = 1)	Pr(ARM(15) = 2|ARM(10) = 1)
regression	N/A	Pr(ARM(5) = 0,1|ARM(0) = 2)	Pr(ARM(10) = 0,1|ARM(5) = 2)	Pr(ARM(15) = 0,1|ARM(10) = 2)
disappearance	N/A	Pr(ARM(5) = 0|ARM(0) = 1)	Pr(ARM(10) = 0|ARM(5) = 1)	Pr(ARM(15) = 0|ARM(10) = 1)

ARM(0), ARM(5), ARM(10) and ARM(15) represent the 3-level ARM severity scale at baseline, 5-year, 10-year and 15-year follow-up, respectively. N/A = not applicable.

### Analyzing transition probabilities separately

This paper presents two different ways for analyzing the transition courses of ARM. We specifically want to draw inferences of the relationship between risk factors and patients' incidence, progression, regression and disappearance probabilities. The first approach is to define different probabilities based on the definitions provided in the previous subsection and analyze each probability separately.

Formally, let *O*_*ij *_be the disease severity scale of the *i*th individual at the *j*th examination (*i *= 1, ⋯ , *N*; *j *= 1, ⋯ ,*J*). In our application, (*O*_*i*1_, *O*_*i*2_, *O*_*i*3_, *O*_*i*4_) represents the collection of the combined 3-level severity scales of ARM for the *i*th individual at baseline, 5-year follow-up, 10-year follow-up and 15-year follow-up.

The possible values of *O*_*ij *_are 0 = disease free, 1 = early stage of the disease, and 2 = late stage of the disease. Suppose Inc_*ij *_is the indicator of incidence for the *i*th individual at the *j*th examination with values

$${\text{Inc}}_{ij}=\{\begin{array}{ll} 1 & \text{if }O_{i(j-1)}=0\text{ and }O_{ij}=1\text{ or }2,\\ 0 & \text{if }O_{i(j-1)}=0\text{ and }O_{ij}=0,\\ \text{NA} & \text{if }O_{i(j-1)}\neq 0, \end{array}$$

where *j *= 2, ⋯ ,*J *and NA represents a missing value. The indicators of progression (Pro_*ij*_), regression (Reg_*ij*_) and disappearance (Dis_*ij*_) for the *i*th individual at the *j*th examination are defined as follows:

$$\begin{array}{c} {\text{Pro}}_{ij}=\{\begin{array}{ll} 1 & \text{if }O_{i(j-1)}=1\text{ and }O_{ij}=2,\\ 0 & \text{if }O_{i(j-1)}=1\text{ and }O_{ij}\neq 2,\\ \text{NA} & \text{if }O_{i(j-1)}\neq 1; \end{array}\\ {\text{Reg}}_{ij}=\{\begin{array}{ll} 1 & \text{if }O_{i(j-1)}=2\text{ and }O_{ij}=0\text{ or }1,\\ 0 & \text{if }O_{i(j-1)}=2\text{ and }O_{ij}=2,\\ \text{NA} & \text{if }O_{i(j-1)}\neq 2; \end{array}\\ {\text{Dis}}_{ij}=\{\begin{array}{ll} 1 & \text{if }O_{i(j-1)}=1\text{ and }O_{ij}=0,\\ 0 & \text{if }O_{i(j-1)}=1\text{ and }O_{ij}\neq 0,\\ \text{NA} & \text{if }O_{i(j-1)}\neq 1. \end{array} \end{array}$$

It should be noted that for each transition course, there are *J *- 1 indicators from the same individual and, therefore, these indicators are correlated.

To model the relationship between, say, incidence and risk factors *x*_*ij*1_, ⋯ , *x*_*ijP*_, we can use a regression analysis for the longitudinal data. Here, we adopt a marginal model [8,9] for this purpose:

(1) $$\log(\frac{\mu_{ij}}{1-\mu_{ij}})=\beta_{0}+\beta_{1}x_{ij1}+\cdots +\beta_{P}x_{ij}P,$$

(2) cov(Inc_*ij*_, Inc_*ik*_) = *f*(*μ*_*ij*_, *μ*_*ik*_; *α*), *j *<*k*,

where *μ*_*ij *_= Pr(Inc_*ij *_= 1) and *f*(·) is a known function. Each transition probability is analyzed separately.

Parameter and standard error estimations can be obtained by the generalized estimating equations (GEE) approach [10,11]. It is worthwhile to point out that, by the definition of the indicator of each transition type, individuals whose indicators are equal to 1 at time *j *will have missing values at time *j *+ 1. When estimating the correlation between two adjacent time points, only those individuals whose indicators are equal to 0 at time *j *are included in the analysis and, therefore, we assume that the correlation among individuals who have indicator values equaling to 1 at time *j *is similar to those who have value 0. Here, we are most interested in inferences of *β*'s in the marginal mean. GEE approach can guarantee the consistency of $\hat{\beta }$'s even if the above equal-correlation-assumption is incorrect [9].

### Analyzing probabilities jointly: the transition model

#### Model

A transition model specifies a generalized linear model for the conditional distribution of the current disease status, given the past responses. To obtain the desired transition probabilities, the transition model used in this study specifies the conditional distribution given on the immediately preceding response.

Then, the proposed transition model is

(3) $$\begin{array}{lll} \log\left\{\frac{\mathrm{Pr}(O_{ij}>c|o_{i(j-1)})}{\mathrm{Pr}(O_{ij}\leq c|o_{i(j-1)})}\right\} & = & \theta_{c}+\gamma_{1c}I(o_{i(j-1)}=1)+\gamma_{2c}I(o_{i(j-1)}=2)\\ & + & \beta_{1c}x_{ij1}+\cdots +\beta_{Pc}x_{ijP}\\ & + & \tau_{11}I(o_{i(j-1)}=1)x_{ij1}+\cdots +\tau_{1P}I(o_{i(j-1)}=1)x_{ijP}\\ & + & \tau_{21}I(o_{i(j-1)}=2)x_{ij1}+\cdots +\tau_{2P}I(o_{i(j-1)}=2)x_{ijP}, \end{array}$$

where *j *= 2, ⋯ , *J*; *c *= 0, 1; *o*_*i*(*j*-1) _is the realization of *O*_*i*(*j*-1)_; and *I*(*o*_*i*(*j*-1) _= *k*) = 1 if *o*_*i*(*j*-1) _= *k *and 0 otherwise, for *k *= 1, 2.

Some key features of the proposed transition model are as follows. First, because the disease severity scale *O*_*ij *_is an ordinal scale, we model the cumulative probability (*O*_*ij *_> *c*) similar to the proportional odds model [12], rather than the category probability (*O*_*ij *_= c). Second, our model allows the regression coefficients *γ*'s and *β*'s to be different for different *c*. We also add the interactions between the preceding response (*I*(*o*_*i*(*j*-1) _= 1), *I*(*o*_*i*(*j*-1) _= 2)) and the risk factors of interest *x*_*ij*1_, ⋯ ,*x*_*ijP*_. These modelling approaches allow the risk factor effects varying with *c *and the disease level at examination *j *- 1. Because different transition probabilities can be obtained by selecting a different *c *and a different disease level at examination *j *- 1, model (3) enables us to investigate the risk factor effects for different transition probabilities. Third, the proposed model has the potential to grow quickly given the possible cutpoints *c *and interactions. To efficiently apply the model, regression coefficients for covariates that are not of major interest and serve as confounding effects may be assumed to be independent of *c *or as having no interactions with the previous disease status.

#### Parameter interpretation

Through the transition model (3), we can derive the relationship of the incorporated risk factors with different transition probabilities. When *c *= 0 and (*I*(*o*_*i*(*j*-1) _= 1), *I*(*o*_*i*(*j*-1) _= 2)) = (0, 0), the conditional probability Pr(*O*_*ij *_> c|*o*_*i*(*j*-1)_) = Pr(*O*_*ij *_= 1 or 2|*o*_*i*(*j*-1) _= 0), which represents the incidence probability.

Therefore,

(4) *β*_*p*0 _= log odds ratio of the disease incidence for every one unit increase in *x*_*ijp*_.

When *c *= 1 and (*I*(*o*_*i*(*j*-1) _= 1), *I*(*o*_*i*(*j*-1) _= 2)) = (1, 0), the conditional probability becomes the progression probability, thus,

(5) (*β*_*p*1 _+ *τ*_1*p*_) = log odds ratio of the disease progression for every one unit increase in *x*_*ijp*_.

When *c *= 1 and (*I*(*o*_*i*(*j*-1) _= 1), *I*(*o*_*i*(*j*-1) _= 2)) = (0, 1), we then have the conditional probability equal to one minus the regression probability, thus,

(6) -(*β*_*p*1 _+ *τ*_2*p*_) = log odds ratio of the disease regression for every one unit increase in *x*_*ijp*_.

When *c *= 0 and (*I*(*o*_*i*(*j*-1) _= 1), *I*(*o*_*i*(*j*-1) _= 2)) = (1, 0), the conditional probability is equal to one minus the disappearance probability, thus,

(7) -(*β*_*p*0 _+ *τ*_1*p*_) = log odds ratio of the disease disappearance for every one unit increase in *x*_*ijp*_.

#### Statistical inference

The likelihood for the *i*th individual can be written as

(8) $$L_{i}(O_{i1},\cdots ,O_{jJ})=\mathrm{Pr}(O_{i1})\prod_{j=2}^{J}\mathrm{Pr}(O_{ij}|H_{ij}),$$

where *H*_*ij *_= {(*O*_*i*1_, ⋯, *O*_*i*(*j*-1)_)} is the history for individual *i *at examination *j*. The transition model only specifies the conditional distribution Pr(*O*_*ij*_|*H*_*ij*_), and the marginal distribution Pr(*O*_*i*1_) is left unspecified. For the ordinal data, the marginal distribution cannot be fully determined by the conditional distributions, and the full likelihood is unavailable. An alternative is to estimate the parameters by maximizing the conditional likelihood [13]

(9) $$L_{\text{cond}}=\prod_{i=1}^{N}\mathrm{Pr}(O_{i2},\cdots ,O_{jJ}|O_{i1})=\prod_{i=1}^{N}\prod_{j=2}^{J}\mathrm{Pr}(O_{ij}|H_{ij}).$$

If the first-order Markov assumption (i.e., *O*_*ij *_is assumed to depend on the past responses only through the immediately preceding response) is correct, the conditional distribution Pr(*O*_*ij*_|*H*_*ij*_) = Pr(*O*_*ij*_|*O*_*i*(*j*-1)_).

Since the transition events {*O*_*ij*_|*O*_*i*(*j*-1)_; *j *= 2, ⋯ ,*J*} are uncorrelated, standard algorithms for fitting the proportional odds models can be used by adding (*I*(*o*_*i*(*j*-1) _= 1), *I*(*o*_*i*(*j*-1) _= 2)) and their interactions with (*x*_*ij*1_, ⋯ , *x*_*ijP*_) as additional covariates.

If the first-order Markov assumption is incorrect, the transition events {*O*_*ij*_|*O*_*i*(*j*-1)_; *j *= 2, ⋯ , *J*} are not independent. However, we still want to model Pr(*O*_*ij*_|*O*_*i*(*j*-1)_) because of the well fitting interpretations for *β*'s and *τ*'s under model (3). Hence, model (3) must be fit by using approaches that can account for the dependency among (*O*_*i*2_, ⋯ , *O*_*iJ*_) given *O*_*i*1_. We adopt the model for analyzing clustered ordinal measurements as proposed by Heagerty and Zeger [11]. In Heagerty and Zeger's model, two regression models are specified: one to describe the marginal means between ordinal outcomes and risk factors, and the other to describe the associations among repeated measurements. When analyzing the transition events, (3) can be viewed as the marginal mean model, and the association model is set as

(10) $$\begin{array}{lll} \log\{\text{OR}[I(O_{ij}>c_{1}),I(O_{ik}>c_{2})|o_{i1}]\} & = & \log\left\{\frac{\mathrm{Pr}(O_{ij}>c_{1},O_{ik}>c_{2})\mathrm{Pr}(O_{ij}\leq c_{1},O_{ik}\leq c_{2})}{\mathrm{Pr}(O_{ij}>c_{1},O_{ik}\leq c_{2})\mathrm{Pr}(O_{ij}\leq c_{1},O_{ik}>c_{2})}\right\}\\ & = & \alpha_{0}+\alpha_{1}I(o_{i1}=1)+\alpha_{2}I(o_{i1}=2), \end{array}$$

where *j *<*k *= 2, ⋯ , *J *and c_1_, c_2 _= 0, 1. The odds ratio between two repeated measurements is assumed to depend on the measurement at time 1. This assumption may be checked and modified, if necessary. The association model may be simplified as an intercept only model or by imposing additional covariates to the model. If none of *α *0, *α *1 and *α *2 are significant, the first-order Markov assumption is appropriate, and we thus recommend to use the standard proportional odds model for inferences to avoid unnecessary complication.

Analysts may choose from three different GEE estimating methods to estimate the parameters in equations (3) and (10) when implementing Heagerty and Zeger's model. First-order GEE (GEE1 – [10]) treats the parameters in the association model (10) as nuisance and is focused primarily on obtaining the parameters in the marginal mean model (3). Second-order GEE (GEE2 – [14]) estimates the parameters in both (3) and (10) jointly. Extended alternating logistic regressions (ALR – [15]) replaces the estimating equation in GEE1 for the parameters in (10) by an unbiased nonlinear estimating equation and offers high efficiency in the estimation of both sets of parameters. The standard errors of all three methods are calculated using robust "sandwich" variance estimators. GEE2 estimates the association parameters in (10) most precisely; however, it has the disadvantages that the consistency of the parameters in (3) depends on having specified the correct model for the association model, and that its computational burden quickly grows to infeasibility as data clusters become large. Thus in situations where inference regarding the parameters in the marginal mean model (3) is primary or when estimation using GEE2 is intractable, GEE1 or ALR may be most appropriate.

It should be noted that the proportional odds model and Heagerty and Zeger's model both make the proportional odds assumption. That is to say, they assume the regression coefficients to be independent of cutpoints *c*. The transition model (3) is more complicated, since the model allows *γ*'s and *β*'s to be different for different *c*. To relax the proportional odds assumption, one can first expand the original input data set for the ordinal outcomes *O*_*ij *_into a new data set for cumulative probability variables (*I*(*O*_*ij *_> 0), *I*(*O*_*ij *_> 1)) plus cutpoint identifiers (*I*(*c *= 0), *I*(*c *= 1)), and then add interactions between the cutpoint identifiers and the covariates. Details for using SAS to implement the "partial" proportional odds model can be found in Chapter 15 of the book by Stokes et al. [16]. For fitting Heagerty and Zeger's model with cutpoint-varied regression coefficients, readers can refer to the article by Huang et al. [17].

### Evaluating equal covariate effects across transition probabilities

The separate analysis allows different covariate effects on different transition probabilities, however, it also risks losing available information and encountering an insufficient sample size. The joint analysis "borrows strength" in part by assuming equality with respect to some confounding effects on transition probabilities, and in certain cases, this may be inappropriate. This section presents an approach for the empirical examination of the equal-confounding-effect assumption, utilizing separate analytical results. Then, the joint transition model can be modified accordingly in order to reduce the complexity of the model.

Suppose that the covariate *x*_*ijp *_is not of major interest and serves as a confounding variable. To evaluate whether *x*_*ijp *_has equal effects on different transition probabilities in the transition model (3), one can test hypotheses *H*_01 _: *β*_*p*1 _= *β*_*p*0_, *H*_02 _: *τ*_1*p *_= 0 and *H*_03 _: *τ*_2*p *_= 0. After fitting the separate models, we obtain the estimated log odds ratios for every one unit increase in *x*_*ijp *_on incidence $\left({\hat{\beta }}_{p}^{\left(I\right)}\right)$, progression $\left({\hat{\beta }}_{p}^{\left(P\right)}\right)$, regression $\left({\hat{\beta }}_{p}^{\left(R\right)}\right)$ and disappearance $\left({\hat{\beta }}_{p}^{\left(D\right)}\right)$. Based on equations (4)-(7), it is reasonable to predict *β*_*p*0_, *β*_*p*1_, *τ*_1*p *_and *τ*_2*p *_for the joint model as

$$\begin{array}{c} {\tilde{\beta }}_{p0}={\hat{\beta }}_{p}^{\left(I\right)},\\ {\tilde{\beta }}_{p1}={\hat{\beta }}_{p}^{\left(I\right)}+{\hat{\beta }}_{p}^{\left(P\right)}+{\hat{\beta }}_{p}^{\left(D\right)},\\ {\tilde{\tau }}_{1p}=-({\hat{\beta }}_{p}^{\left(I\right)}+{\hat{\beta }}_{p}^{\left(D\right)}),\\ {\tilde{\tau }}_{2p}=-({\hat{\beta }}_{p}^{\left(I\right)}+{\hat{\beta }}_{p}^{\left(P\right)}+{\hat{\beta }}_{p}^{\left(R\right)}+{\hat{\beta }}_{p}^{\left(D\right)}). \end{array}$$

Their variance estimators cannot be derived easily because they involve estimations of the covariances between estimators from different models. We propose to estimate the distributions of $({\tilde{\beta }}_{p1}-{\tilde{\beta }}_{p0}),{\tilde{\tau }}_{1p}$ and ${\tilde{\tau }}_{2p}$ using the bootstrap method [18]. It must be noted that in order to perform bootstrapping for repeated measures on each individual, each subject is sampled with replacement rather than individual observations.

Reject, for example, *H*_01 _: *β*_*p*1 _= *β*_*p*0 _at the significance level of *α *if the bootstrap percentile confidence interval of (*β*_*p*1 _- *β*_*p*0_),

$$\left[{\left({\tilde{\beta }}_{p1}-{\tilde{\beta }}_{p0}\right)}_{\alpha /2}^{*},{\left({\tilde{\beta }}_{p1}-{\tilde{\beta }}_{p0}\right)}_{1-\alpha /2}^{*}\right],$$

does not cover 0, where ${\left({\tilde{\beta }}_{p1}-{\tilde{\beta }}_{p0}\right)}_{\alpha /2}^{*}$ is the lower 100(*α*/2)th percentile of the bootstrap replications of statistics $\left({\tilde{\beta }}_{p1}-{\tilde{\beta }}_{p0}\right)$.

In the case where there are many confounders to be tested for the equal-effect assumption, we recommend that each potential confounder is considered separately. In other words, perform bootstrapping for the separate analysis with major risk factors plus one confounder at a time to determine the modelling of this confounder in the transition model.

Three null hypotheses *H*_01_, *H*_02 _and *H*_03 _should be checked separately. If only part of the three null hypotheses are rejected, this means that the covariate effects on various transition probabilities are similar to some extent, and that only corresponding interactions are added. For example, if only *H*_02 _: *τ*_1*p *_= 0 is rejected, the interaction *I*(*o*_*i*(*j*-1) _= 1)*x*_*ijp *_is included.

The proposed procedure for checking the equal-confounding-effect assumption is "empirical", compared with the backward elimination starting at the "full" transition model (i.e., all risk factor effects varying with *c *and the disease level of the previous examination). However, the full transition model is usually too complicated to converge, making the backward elimination procedure not feasible.

## Results

The analysis we report here aims to examine whether a birth cohort effect is observed for ARM. The birth cohort effect is defined as the variation in developing ARM that arises from the different exposures to each birth cohort. Thus, if a birth cohort effect exists, individuals from different birth cohorts would have different chances of developing ARM, even if they are of the same age. The birth cohort effect on the prevalence of ARM has been investigated elsewhere [19]. Here, we focus on the birth cohort effect on different transition probabilities

### Analytical methods

To graphically display the observed birth cohort patterns, we first aggregated the data into a two-way table by birth year and age group in 5-year intervals, and calculated different transition probabilities of ARM in each cell. Next, we plotted the transition probability against age for each birth cohort. For our application, 9 birth cohorts and 10 age groups were constructed (birth cohorts: ≤1907, 1908–1912, 1913–1917, 1918–1922, 1923–1927, 1928–1932, 1933–1937, 1938–1942, ≥1943; age groups: ≤49, 50–54, 55–59, 60–64, 65–69, 70–74, 75–79, 80–84, 85–89, ≥90).

The approaches proposed in the previous sections were used to analyze the transition probabilities separately and jointly, in order to provide significance tests of birth cohort effects. The model for the separate analysis of incidence is as follows:

(11) $$\log(\frac{\mu_{ij}}{1-\mu_{ij}})=\beta_{0}+\beta_{1}[{\left(\text{age in }1987\right)}_{i}-65]+\beta_{2}({\text{age}}_{ij}-65)+\beta_{3}{\left(confounders\right)}_{ij},$$

(12) var(Inc_*ij*_) = *μ*_*ij*_(1 - *μ*_*ij*_) and corr(Inc_*ij*_, Inc_*ik*_) = *α*_0_,

where *μ*_*ij *_= Pr(Inc_*ij *_= 1), *j *<*k *= 2: 5-year follow-up; 3: 10-year follow-up; 4: 15-year follow-up, (age in 1987)_*i *_is the *i*th participant's age in 1987, age_*ij *_is the age of participant *i *at examination *j*, and **(confounders)**_*ij *_represents characteristics that could potentially influence the relationship among ARM, birth cohort and age at the examination, including gender, smoking status, history of heavy drinking, multi-vitamin use, cholesterol level and hypertension status [19] (the boldface type denotes multiple factors). Treatment of ARM is not included as a confounding variable because, at present, there are few medical interventions that have been shown to prevent the incidence or progression of ARM [20,21]. Although surgical intervention in some cases prevents further loss of vision, it usually does not restore vision in the patient. In our Beaver Dam Eye study, no significant relationships were found between the most commonly used interventions and 5-year and 10-year incidences of early or late ARM [20,21]. The concomitant low frequency of use of medication, surgery, and of incidence of early and late ARM limits our ability to detect any meaningful relationship.

The birth cohort effect exp(5*β*_1_) is the odds ratio of ARM incidence for every 5-year decrease in birth year (5-year older birth cohort) among people with the same age. The age effect exp(5*β*_2_) is the odds ratio for every 5-year increase in age, comparing people from the same birth cohort. These two effects are adjusted for the identified confounding effects. Here, we chose the "exchangeable" working correlation because the focus was on the birth cohort effect and a reasonable and simple association model (12) was all we needed. The indicator Inc_*ij *_was replaced by Pro_*ij*_, Reg_*ij *_or Dis_*ij *_when analyzing different transition courses.

Before conducting the joint analysis, we evaluated the equal-effect hypotheses *H*_01_, *H*_02 _and *H*_03 _on each of the identified confounding variables in order to reduce the complexity of the model. If the 80% bootstrap percentile confidence interval (with 500 bootstrap replicates) covered 0, the corresponding hypothesis was accepted and the modelling of the confounding variable in the transition model (3) was modified accordingly.

To perform the joint analysis, we fit the following transition model

(13) $$\begin{array}{lll} \log\left\{\frac{\mathrm{Pr}(O_{ij}>c|o_{i(j-1)})}{\mathrm{Pr}(O_{ij}\leq c|o_{i(j-1)})}\right\} & = & \theta_{c}+\gamma_{1c}I(o_{i(j-1)}=1)+\gamma_{2c}I(o_{i(j-1)}=2)\\ & + & \beta_{1c}[{\left(\text{age in }1987\right)}_{i}-65]+\beta_{2c}({\text{age}}_{ij}-65)\\ & + & \tau_{11}I(o_{i(j-1)}=1)[{\left(\text{age in }1987\right)}_{i}-65]+\tau_{12}I(o_{i(j-1)}=1)({\text{age}}_{ij}-65)\\ & + & \tau_{21}I(o_{i(j-1)}=2)[{\left(\text{age in }1987\right)}_{i}-65]+\tau_{22}I(o_{i(j-1)}=2)({\text{age}}_{ij}-65)\\ & + & g({\left(confounders\right)}_{ij}), \end{array}$$

where *c *= 0, 1, *j *= 2, 3, 4 and the function *g*(·) depends on the significance of hypotheses *H*_01_, *H*_02 _and *H*_03 _for each of the identified confounding variables. We added (10) as the association model and fit a Heagerty and Zeger's model with cutpoint-varied regression coefficients. Because our focus was not on the degree of association among the transition events {*O*_*ij*_|*O*_*i*(*j*-1)_; *j *= 2, ⋯ , *J*_*i*_}, we used GEE1 as the estimating method, which is robust to the misspecification of the association model (10). The birth cohort effects of ARM incidence, progression, regression and disappearance are exp(5*β*_10_), exp{5(*β*_11 _+ *τ*_*11*_)}, exp{-5(*β*_11 _+ *τ*_21_)} and exp{-5(*β*_10 _+ *τ*_11_)}, respectively. The age effects are exp(5*β*_20_), exp{5(*β*_21 _+ *τ*_12_)}, exp{-5(*β*_21 _+ *τ*_22_)} and exp{-5(*β*_20 _+ *τ*_12_)} for ARM incidence, progression, regression and disappearance, respectively.

### Results

**Figure 2:**
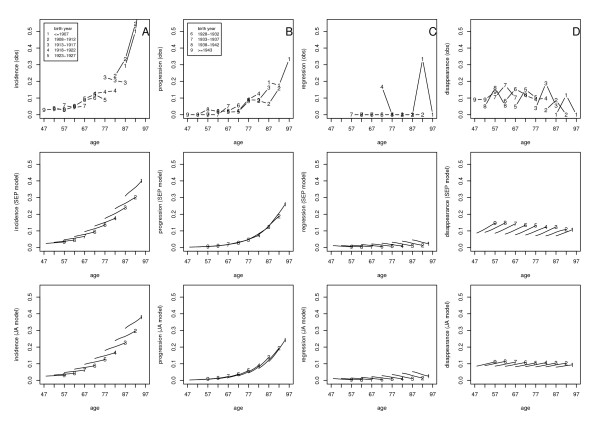
**Relation of age to ARM transition probabilities for different birth cohorts**. A includes plots for incidence probabilities, B includes plots for progression probabilities, C includes plots for regression probabilities, and D includes plots for disappearance probabilities. Within each transition probability, the top panel is the plot based on observed data (obs), the middle panel is the plot based on separate analysis (SEP model), and the bottom panel is the plot based on joint analysis (JA model). In each panel, different numbers represent different birth cohorts (birth years).

The incidence, progression, regression and disappearance probabilities of ARM were: at the 5-year follow-up: 88, 41, 24 and 66 per 1,000 individuals; at the 10-year follow-up: 83, 48, 30 and 141 per 1,000 individuals; and at the 15-year follow-up: 78, 79, 0 and 92 per 1,000 individuals, respectively. Panels in the first row of Figure 2 show the different observed ARM transition probabilities versus age for different birth cohorts. For ARM incidence and progression, we observed that as people became older, the chances of developing the corresponding transition events increased. Those in the older birth cohorts tended to have a higher probability of developing ARM incidence events than those in younger cohorts, even if they had the same age, suggesting a birth cohort effect on the ARM incidence. A birth cohort effect was not as apparent for progression as it was for incidence. The regression probabilities were equal to zero in most of the birth cohorts, making it difficult to judge the birth cohort effect. When comparing people from the same birth cohort, the disappearance probabilities increased and then decreased when the age increased. The younger birth cohort seems to have a positive effect on the ARM disappearance but the trend is not clear.

Table 2 contains the 80% bootstrap percentile confidence intervals for testing the equal-effect hypotheses *H*_01_, *H*_02 _and *H*_03 _on identified confounding variables. None of the confounding variables reject the hypotheses, thus we can assume that the regression coefficients for these confounders are independent of *c *and that there are no interactions with the previous response in model (13). That is to say:

**Table 2:** Bootstrap percentile confidence intervals

confounding variables/hypotheses	*H*_01 _: *β*_*p*1 _= *β*_*p*0_	*H*_02 _: *τ*_1*p *_= 0	*H*_03 _: *τ*_2*p *_= 0
male gender	(-0.61, 0.56)	(-0.40, 0.44)	(-0.61, 0.68)
pack years smoked	(-0.013, 0.0087)	(-0.0065, 0.0096)	(-0.0096,0.014)
past heavy drinker	(-0.87, 0.71)	(-0.50, 0.57)	(-0.70, 0.94)
current heavy drinker	(-78.32, 1.54)	(-1.17, 40.37)	(-1.62, 78.67)
past vitamin user	(-0.71, 0.71)	(-0.56, 0.52)	(-0.88, 0.75)
current vitamin user	(-0.65, 0.71)	(-0.45, 0.49)	(-0.78, 0.68)
total cholesterol	(-0.0059, 0.0053)	(-0.0048, 0.0046)	(-0.0063, 0.0065)
hypertensive	(-0.51, 0.50)	(-0.39, 0.39)	(-0.54, 0.55)

The bootstrap percentile confidence intervals are for testing equal-effect assumptions. The confidence intervals shown in the table are the 80% bootstrap percentile confidence interval.

(14) *g*(**(confounders)**_*ij*_) = ***β***_3 _× **(confounders)**_*ij*_.

It should be noted that the bootstrap confidence interval for "current heavy drinker" is very wide, compared to other variables. This is caused by the large standard error of its regression coefficient estimate in modelling the disappearance probabilities. Only 0.9% of current drinkers had experienced the disappearance events. We performed a separate analysis for disappearance with and without "current heavy drinker" and obtained results that were similar for other variables in the model. To be comparable with our previous results, we decided to keep "current heavy drinker" in the model.

The fitted lines of transition probabilities over age by birth cohort based on the separate analysis (11, 12) are shown in the panels of the second row of Figure 2. The fitted lines were obtained by smoothing the estimated probabilities of the transition event versus the age for each birth cohort. The third row of Figure 2 represents the fitted transition probabilities based on the transition model (13, 14). Model (10) was first used as the association model, but because both *α*_1 _and *α*_2 _were not significant, we simplified the association model as

(15) log{OR[*I*(*O*_*ij *_> *c*_1_), *I*(*O*_*ik *_> *c*_2_)|*O*_*i*1_]} = *α*_0_,

and obtained ${\hat{\alpha }}_{0}=-0.97$ (95% CI: -1.48, -0.46). For all four transition probabilities, the results from the two approaches were pretty close and they fit the data equally well.

**Figure 3:**
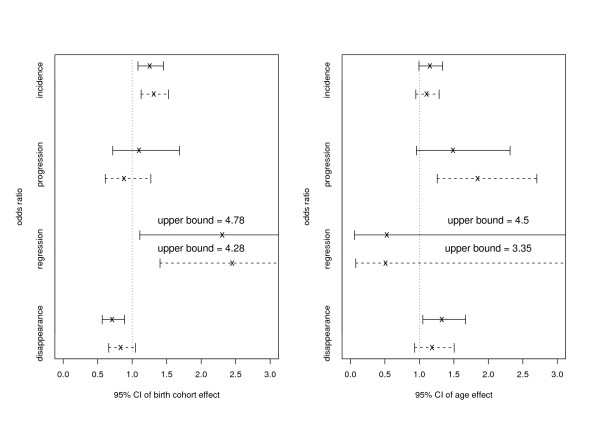
**Confidence intervals of birth cohort and age effects on ARM**. Both birth cohort and age effects are represented by the odds ratio. Both effects are adjusted for gender, smoking status, history of heavy drinking, multi-vitamin use, cholesterol level, and hypertension. In each panel, solid lines are fitted from the models (11, 12) for separate analysis and dashed lines are from the joint analysis models (13, 14, 15). From left to middle to right, each "line segment" displays lower 95% confident interval (CI), estimate and upper 95% CI.

Figure 3 shows the birth cohort and age effects on various ARM transition events. Controlling for age and other risk factors, the participants from the older birth cohorts were more likely to develop ARM incidence than those from the five-year younger cohort. Within the same birth cohort, aging increased the chance of developing ARM progression. There were significant birth cohort effects on ARM regression (the older the birth cohort, the more likely the ARM). The separate analysis revealed that the younger birth cohort and the older age had a positive effect on ARM disappearance; however, the joint analysis did not find these two effects significant. It should be noted that the estimated effects on the regression probability from the transition model (13, 14, 15) had much narrower CI's than those from the separate approach. This might explain the power gained in the joint analysis.

To evaluate the impact of the first-order Markov assumption on the joint analysis, we had fit a standard proportional odds model to models (13, 14). Results can be found from Additional files 1 and 2. In summary, approaches with and without the first-order Markov assumption provided consistent parameter estimates, but this Markov assumption resulted in much wider CI's for birth cohort and age effects. These reflected the robustness of the regression coefficients in (3) for the misspecification of the association model (10) and the power gained from an appropriate association model.

## Discussion

In this paper, we define regression and disappearance as Reg_*ij *_and Dis_*ij *_in Table 1 and in the methods section. The definitions for these two transition courses are not very desirable. Therefore it may be more desirable to define the regression as:

$${\text{Reg}}_{ij}^{*}=\{\begin{array}{ll} 1 & \text{if }O_{i(j-1)}=2\text{ and }O_{ij}=1,\\ 0 & \text{if }O_{i(j-1)}=2\text{ and }O_{ij}\neq 1,\\ \text{NA} & \text{if }O_{i(j-1)}\neq 2; \end{array}$$

and the disappearance as:

$${\text{Dis}}_{ij}^{*}=\{\begin{array}{ll} 1 & \text{if }O_{i(j-1)}=1\text{ or 2 and }O_{ij}=0,\\ 0 & \text{if }O_{i(j-1)}=1\text{ or 2 and }O_{ij}\neq 0,\\ \text{NA} & \text{if }O_{i(j-1)}=0. \end{array}$$

We select Reg_*ij *_and Dis_*ij *_for two reasons. First, they are the direct result of the transition model (3). The proposed transition model models (*I*(*O*_*ij *_> 0), *I*(*O*_*ij *_> 1)) (cumulative probabilities of the current response) and (*I*(*o*_*i*(*j*-1) _= 1), *I*(*o*_*i*(*j*-1) _= 2)) (level indicators of the preceding response). This modelling can result in the incidence and progression that meet our desired definitions, but not those of regression and disappearance. Since our motivational example was more interested in incidence and progression than in the other two courses, we thus adopted the above modelling. Second, the selected regression and disappearance are very close to the desired ${\text{Reg}}_{ij}^{*}$ and ${\text{Dis}}_{ij}^{*}$ in our ARM application. Because late ARM was rare (Figure 1), Dis_*ij *_was close to ${\text{Dis}}_{ij}^{*}$ Also, none of the people with late ARM became disease free in the follow-up, and Dis_*ij *_was equal to ${\text{Dis}}_{ij}^{*}$.

To obtain the inference for ${\text{Dis}}_{ij}^{*}$, one can replace the level indicators of the preceding response with cumulative probabilities (*I*(*o*_*i*(*j*-1) _> 0), *I*(*o*_*i*(*j*-1) _> 1)) in model (3) and set *c *= 0 and (*I*(*o*_*i*(*j*-1) _> 0), *I*(*o*_*i*(*j*-1) _> 1)) = (1, 1). If the regression ${\text{Reg}}_{ij}^{*}$ is of interest, then we can use the indicators of the current response (*I*(*Oij *= 1), *I*(*O*_*ij *_= 2)) as dependent variables and fit a linear generalized logit model [22], setting *c *= 1 and (*I*(*o*_*i*(*j*-1) _= 1), *I*(*o*_*i*(*j*-1) _= 2)) = (0, 1). Analysts can select modelling strategies for current and past responses based on interested transition probabilities, then modify the definitions of secondary transition probabilities accordingly, the same as we did for the ARM birth cohort study. Or, one could fit several different transition models with different modelling selections and draw inferences for interested transition probabilities from corresponding models.

This paper considered two different approaches for analyzing longitudinal disease staging data. In the separate analysis, the incidence, progression, regression and disappearance probabilities are marginally defined, modelled and estimated. One can easily modify the definition of a transition probability to accommodate various needs (e.g., using ${\text{Reg}}_{ij}^{*}$ and ${\text{Dis}}_{ij}^{*}$ for analysis). The separate analysis also allows different covariate effects on different transition probabilities, which is best for carefully describing specific precursor effects on transition probabilities and provides an excellent reference for checking the assumptions on which the transition model relies. In contrast, a joint transition model can borrow strength from all transition probabilities. For confounding variables that do not show different effects on different transition probabilities through the examination of separate analytical results, the transition model can adopt the equal-effect assumption to reduce the complexity of the model. One limitation is its inflexibility in simultaneously obtaining desirably defined transition probabilities as described in the above discussion. As a general strategic recommendation: It is natural to first analyze each transition probability separately for initial findings and empirical examination of the equal-confounding-effect assumption. Then, the transition model, taking separate analytical results into account, is useful to refine and clarify those outcomes that are indecisive in separate analysis.

The transition model (3) can potentially grow very large, with increasing number of levels, covariates and follow-ups. To ensure a large enough sample size for implementing the model, one can examine the cross tabulations of *O*_*ij *_versus *O*_*i*(*j*-1) _for *j *= 2, ⋯ , *J*, stratifying by possible values of major risk factors. It is recommended that no cell value should be less than 5.

There are many possible generalizations of the proposed framework. Generalization to allow a disease severity scale with more than three levels can be easily done. However, with more than three disease-severity levels the definitions of distinct transition probabilities are not trivial, thus researchers may need to first define the transition probabilities according to the study aims and then work on the modelling of current and past responses to meet those aims. Also, the proposed approaches may be generalized to allow subjects to be measured at different sets of times (i.e., unequally-spaced follow-up). The transition model (3) solely depends on the immediately preceding response and, by treating the correlation as nuisance, the association model (10) is taken to handle the inter-correlation among the transition events {*O*_*ij*_|*O*_*i*(*j*-1)_; *j *= 2, ⋯ , *J*_*i*_}. Thus, the model does not result in different interpretations of regression coefficients in (3) for subjects with different numbers of examinations, as discussed in [8]. In the case where additional subjects can be recruited at any time points during the study (i.e., an open population), these newly recruited samples will have missing disease severity observations at time points before their recruitment. If their missingness is completely at random [23], then the situation can be handled by only including collected examinations and their associated covariates.

## Conclusion

This paper proposed and demonstrated a framework for studying the relationship of disease incidence, progression, regression and regression with risk factors of interest. Our proposed framework includes two different analytical approaches. One approach can define, model and estimate the relationship between each transition probability and risk factors separately. The other approach specifies a transition/conditional probability model to formulate the probability of the current disease level based upon the previous level. It studies the disease as a whole and uses the whole population to estimate these probabilities together. We recommend that one first analyzes each transition probability separately for data exploration and assumption evaluation, and then utilize the transition model to refine and clarify the results. The results of the ARM data analysis show that the parallel application of separate and joint analyses is superior over any in isolation. In this regard, mutually cohesive findings generally will comprise stronger scientific evidence than those supported by only one of the analytical approaches. The fitting methods for the transition model are readily implementable in available software.

## Competing interests

The author declares that they have no competing interests.

## Authors' contributions

GHH formulated the original concept, performed the statistical analysis, interpreted the results and drafted the manuscript.

## Pre-publication history

The pre-publication history for this paper can be accessed here:



## Supplementary Material

Additional file 1 **Joint analysis with the first-order Markov assumption: relation of age to ARM transition probabilities for different birth cohorts**. Clockwise from top left, panels describe incidence, progression, disappearance and regression probabilities. In each panel, black lines are based on observed data, red lines are fitted from the models (11, 12) for separate analysis, green lines are from the joint analysis models (13, 14) under the first-order Markov assumption, and purple lines are from the joint analysis models (13, 14, 15) without the first-order Markov assumption. Also, in each panel, the different numbers represent different birth cohorts (birth years). Click here for file

Additional file 2 **Joint analysis with the first-order Markov assumption: confidence intervals of birth cohort and age effects on ARM**. Both birth cohort and age effects are represented by the odds ratio. Both effects are adjusted for gender, smoking status, history of heavy drinking, multi-vitamin use, cholesterol level, and hypertension. In each panel, red lines are fitted from the models (11, 12) for separate analysis, green lines are from the joint analysis models (13, 14) under the first-order Markov assumption, and purple lines are from the joint analysis models (13, 14, 15) without the first-order Markov assumption. From left to middle to right, each "line segment" displays lower 95% confident interval (CI), estimate and upper 95% CI. Click here for file
