# Supplementary figures and images for: Integrated analysis of incidence, progression, regression and disappearance probabilities
Source: BMC Med Res Methodol. 2008 Jun 25;8:40. doi: 10.1186/1471-2288-8-40 (PMC2459200; doi:10.1186/1471-2288-8-40)

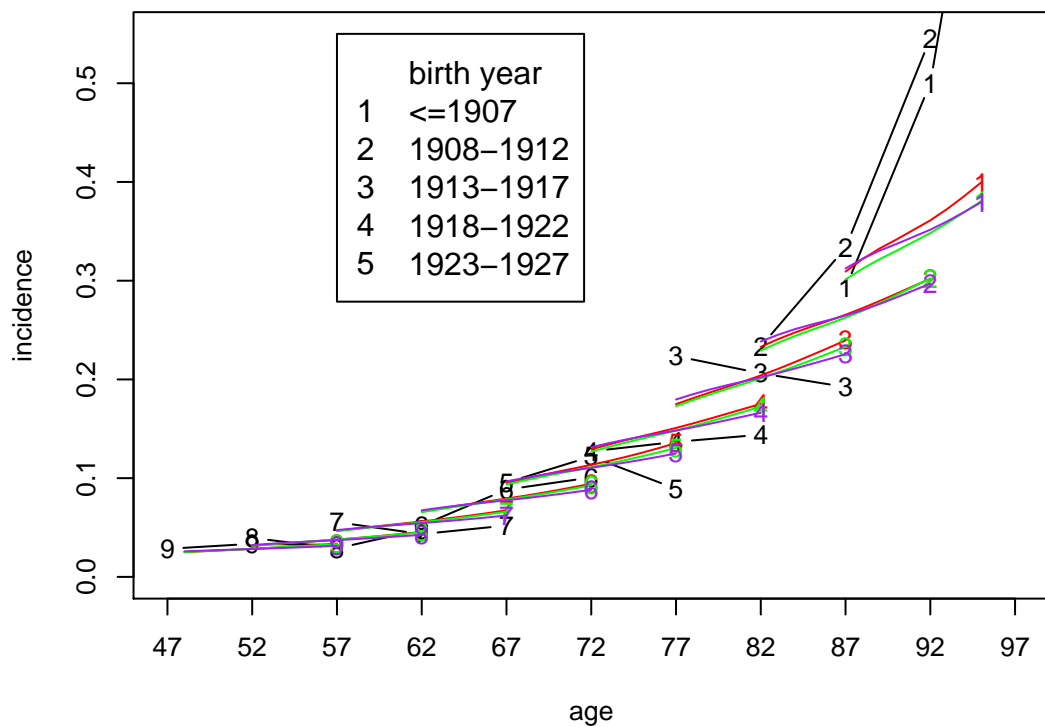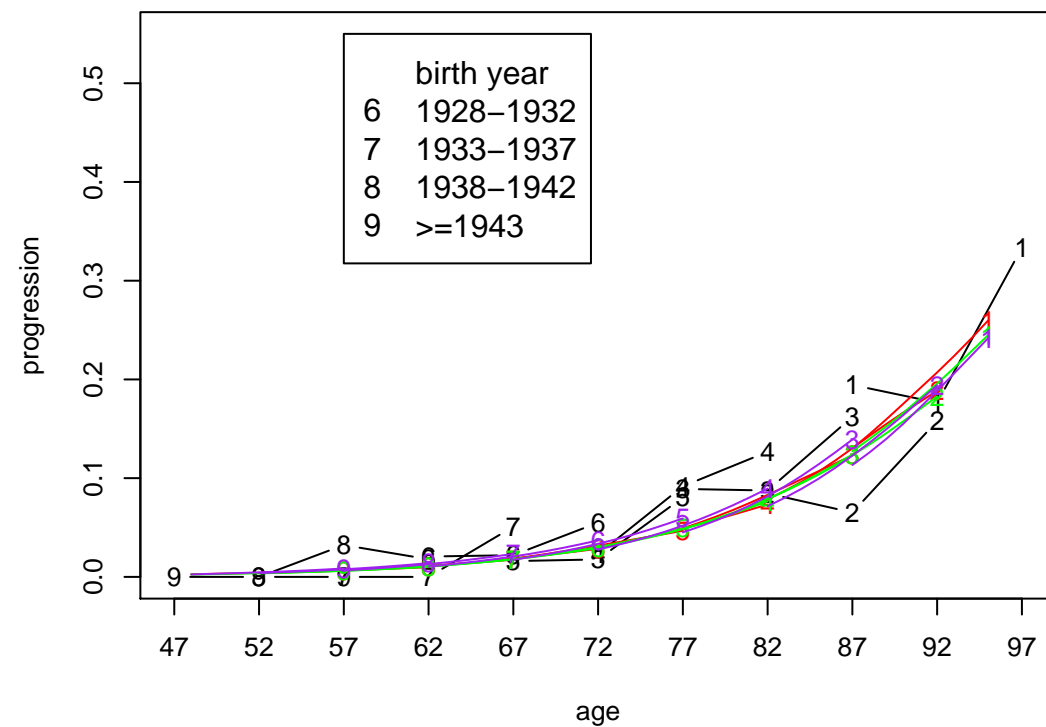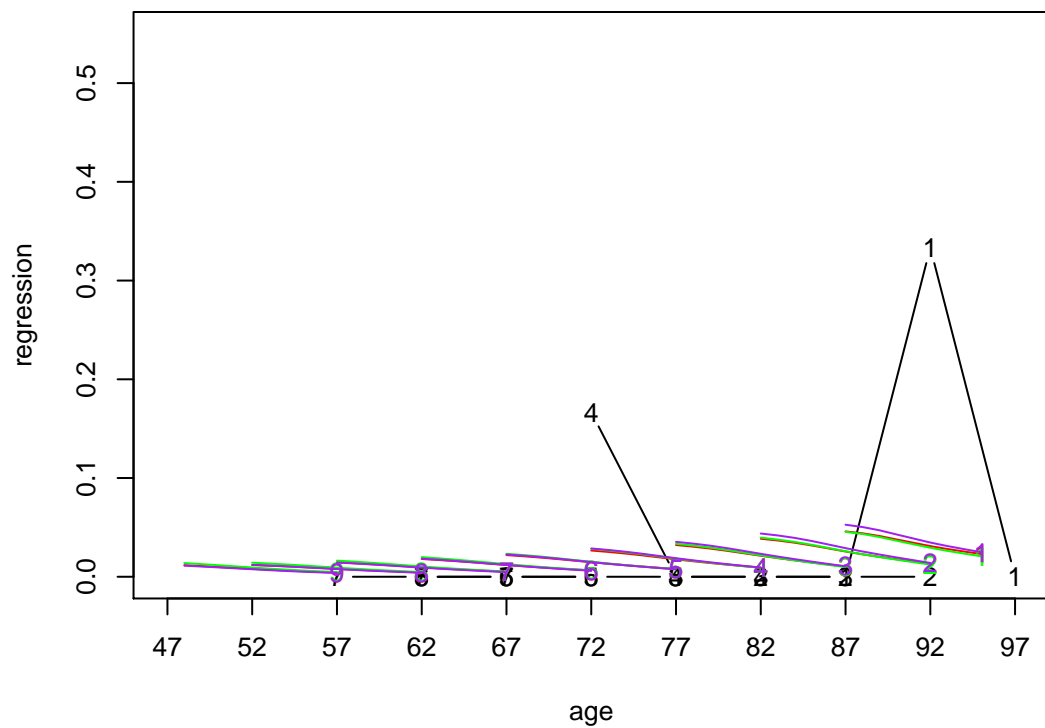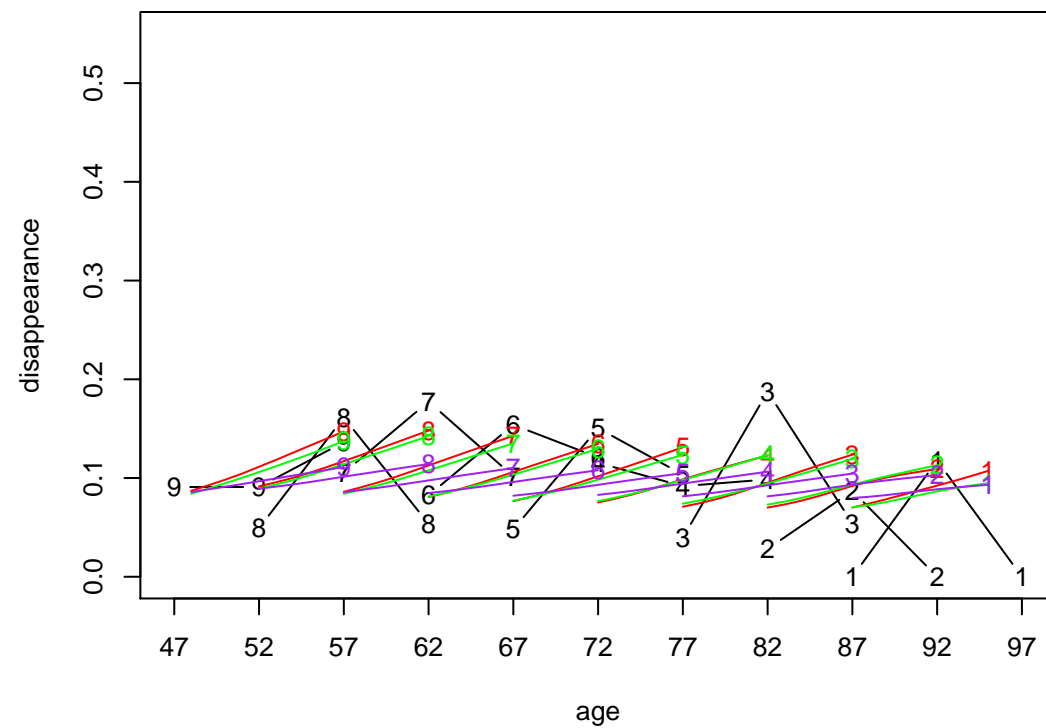

Supplement: Additional file 1 — Joint analysis with the first-order Markov assumption: relation of age to ARM transition probabilities for different birth cohorts. Clockwise from top left, panels describe incidence, progression, disappearance and regression probabilities. In each panel, black lines are based on observed data, red lines are fitted from the models (11, 12) for separate analysis, green lines are from the joint analysis models (13, 14) under the first-order Markov assumption, and purple lines are from the joint analysis models (13, 14, 15) without the first-order Markov assumption. Also, in each panel, the different numbers represent different birth cohorts (birth years). [file 1471-2288-8-40-S1.pdf]

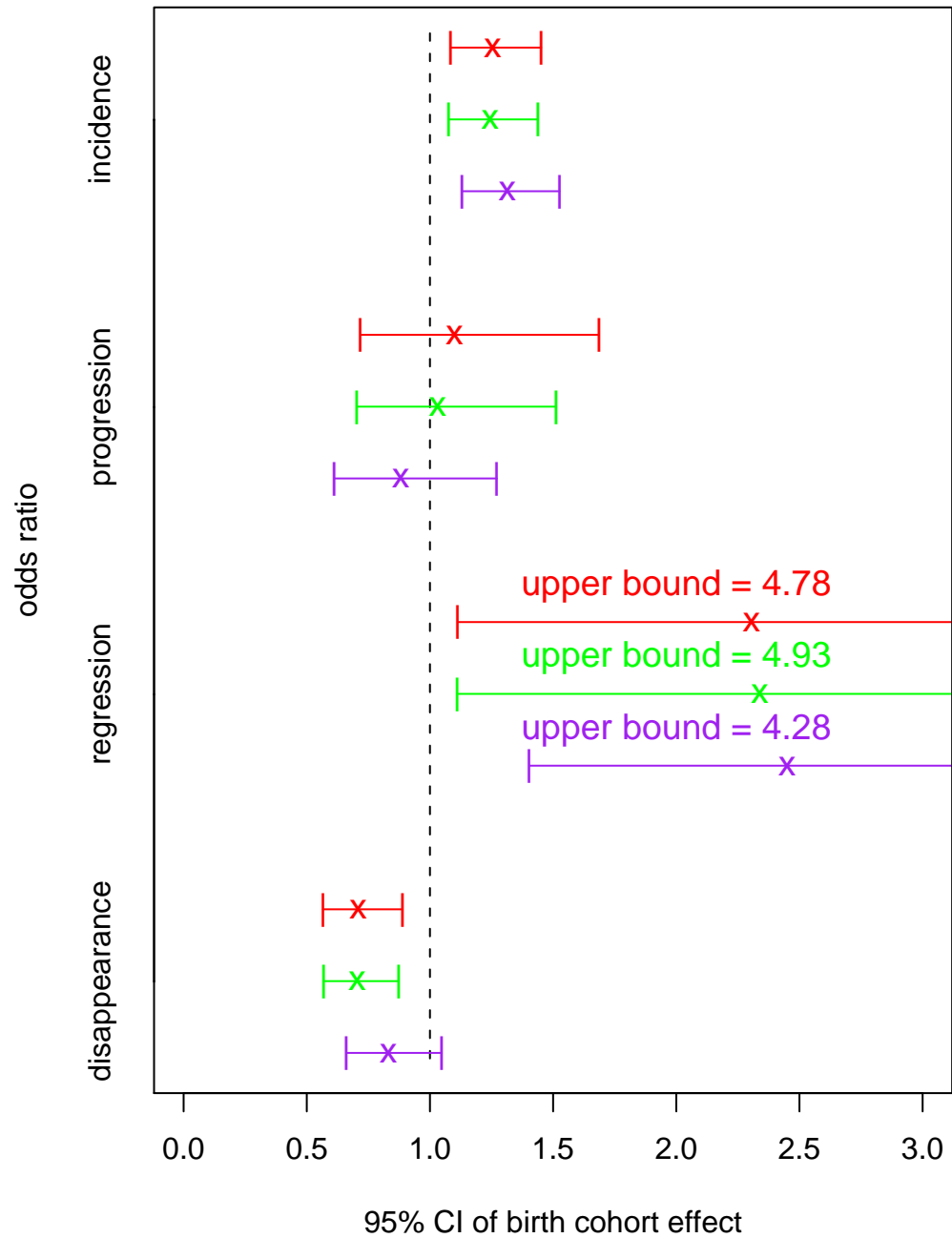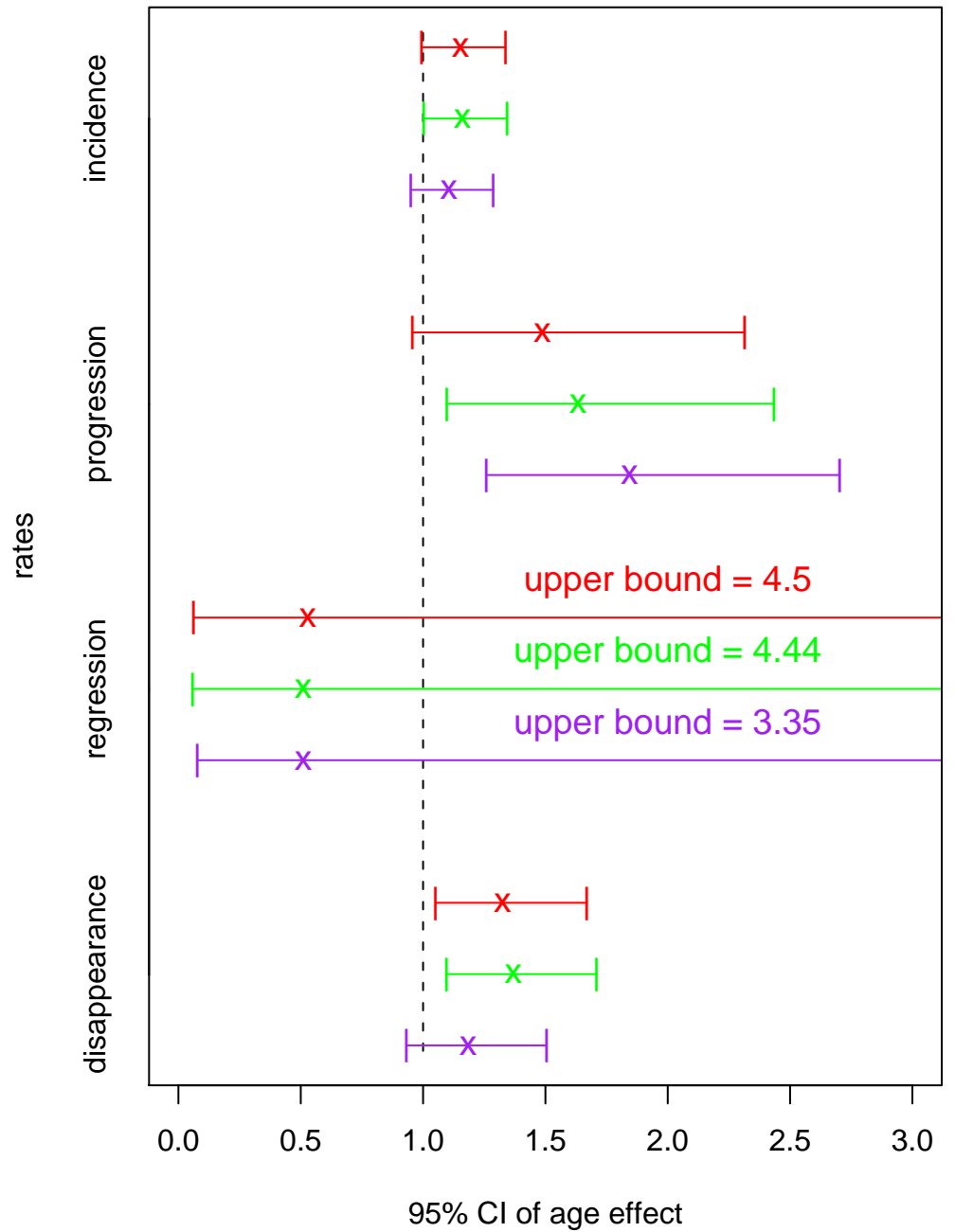

Supplement: Additional file 2 — Joint analysis with the first-order Markov assumption: confidence intervals of birth cohort and age effects on ARM. Both birth cohort and age effects are represented by the odds ratio. Both effects are adjusted for gender, smoking status, history of heavy drinking, multi-vitamin use, cholesterol level, and hypertension. In each panel, red lines are fitted from the models (11, 12) for separate analysis, green lines are from the joint analysis models (13, 14) under the first-order Markov assumption, and purple lines are from the joint analysis models (13, 14, 15) without the first-order Markov assumption. From left to middle to right, each "line segment" displays lower 95% confident interval (CI), estimate and upper 95% CI. [file 1471-2288-8-40-S2.pdf]
